# Supplementary material for: Structural and functional insights into the interaction between Ku70/80 and Pol X family polymerases in NHEJ
Source: Nat Commun. 2025 May 6;16:4208. doi: 10.1038/s41467-025-59133-2 (PMC12056208; doi:10.1038/s41467-025-59133-2)
Supplement: Supplementary file 1 — Supplementary Information [file 41467_2025_59133_MOESM1_ESM.docx]

**Supplementary figures**

**Supplementary Table 1:** Cryo-EM data parameters and statistics

**Supplementary Table 2:** Oligonucleotides (DNA linkers and PCR primers).

| **gRNA-GF-F** | agatCAACGTACGGTCTGATATGCAA |
| --- | --- |
| **gRNA-GF-R** | aaaaTTGCATATCAGACCGTACGTTG |
| **gRNA-LigIV-F** | caccGTTCAGCACTTGAGCAAAAG |
| **gRNA-LigIV-R** | aaacCTTTTGCTCAAGTGCTGAAC |
| **gRNA-PAXX-F** | caccgTGACCGACGCCGCGGAGCTT |
| **gRNA-PAXX-R** | aaacAAGCTCCGCGGCGTCGGTCAc |
| **gRNA-PRKDC-F** | caccGGTACCCACCCAGCACCGCG |
| **gRNA-PRKDC-R** | aaacCGCGGTGCTGGGTGGGTACC |
| **gRNA-POLL-F** | caccgTTCACCAGCTGAGCACCCGG |
| **gRNA-POLL-R** | aaacCCGGGTGCTCAGCTGGTGAAc |
| **gRNA-POLM-F** | caccGATGGAAGAGACCTCAGCAG |
| **gRNA-POLM-R** | aaacCTGCTGAGGTCTCTTCCATC |
| **gRNA-XLF-F** | caccGGAGATTATCCAAATGACAG |
| **gRNA-XLF-R** | aaacCTGTCATTTGGATAATCTCC |
| **gRNA-XRCC5-F** | caccgAGGATCTTACAAGAATTGCA |
| **gRNA-XRCC5-R** | aaacTGCAATTCTTGTAAGATCCTc |
| **HF-Puro-F** | GCCTCGAGGTTTAAACTACGGgatcTCCGCcATGACaGAGTACAAGCCaACaGTG |
| **HF-Puro-R** | CCGCATGTTAGCAGACTTCCTCTGCCCTCGGCACCtGGCTTtCtGGTCATGCACC |
| **Kpn2-AX-Mlu-F** | ccggTCAGGATCTGGTAGCGGTTCCGGATCTCCTAGGTCACCCGGGTCTA |
| **Kpn2-AX-Mlu-R** | cgcgTAGACCCGGGTGACCTAGGAGATCCGGAACCGCTACCAGATCCTGA |
| **kpn2-T2A-Mlu-F** | CCGGAGGGCAGAGGAAGTCTGCTAACATGCGGTGACGTCGAGGAGAATCCTGGACCCGGGtcactcA |
| **kpn2-T2A-Mlu-R** | CGCGTgagtgaCCCGGGTCCAGGATTCTCCTCGACGTCACCGCATGTTAGCAGACTTCCTCTGCCCT |
| **Ku70-Kpn2-F** | ctctcgTCCGGAGCCACCATGTCAGGGTGGGAGTCATATTACAAAACC |
| **Ku70-Bcu-R** | ctcgtcACTAGTTCAGTCCTGGAAGTGCTTGGTGAGGGCTTC |
| **Ku70-R301A-F** | GAAAACCAAGACCgcGACCTTTAATACAAGTACAGGCGGTTTGCT |
| **Ku70-R301A-R** | CTTGTATTAAAGGTCgcGGTCTTGGTTTTCACTGGTTCATTTGT |
| **Ku70-F303G-F** | CCAAGACCCGGACCggTAATACAAGTACAGGCGGTTTGCTTCTGC |
| **Ku70-F303G-R** | GTACTTGTATTAccGGTCCGGGTCTTGGTTTTCACTGGTTCATTT |
| **Ku70-T307A-F** | CGGACCTTTAATACAAGTgCAGGCGGTTTGCTTCTGCCTAGCGATACC |
| **Ku70-T307A-R** | GAAGCAAACCGCCTGcACTTGTATTAAAGGTCCGGGTCTTGGTTTTCAC |
| **Ku70-L310G-F** | CCTTTAATACAAGTACAGGCGGTggGCTTCTGCCTAGCGATACCAAGAGGTC |
| **Ku70-L310G-R** | CTAGGCAGAAGCccACCGCCTGTACTTGTATTAAAGGTCCGGGTCTTGG |
| **Ku70-L310R-F** | CCTTTAATACAAGTACAGGCGGTcgGCTTCTGCCTAGCGATACCAAGAGGTC |
| **Ku70-L310R-R** | CTAGGCAGAAGCcgACCGCCTGTACTTGTATTAAAGGTCCGGGTCTTGG |
| **Ku80-R292A-F** | GAAGATATACAAAAAGcAACAGTTTATTGCTTAAATGATGATGATGAAAC |
| **Ku80-R292A-R** | GCAATAAACTGTTgCTTTTTGTATATCTTCTTTTTTTAGGGTTTTTGC |
| **Ku80-D301A-F** | GCTTAAATGATGATGcTGAAACTGAAGTTTTAAAAGAGGATATTATTCAAG |
| **Ku80-D301A-R** | TAAAACTTCAGTTTCAgCATCATCATTTAAGCAATAAACTGTTTCTTTTTG |
| **Ku80-E304A-F** | GATGATGATGAAACTGcAGTTTTAAAAGAGGATATTATTCAAGGGTTCCGC |
| **Ku80-E304A-R** | CCTCTTTTAAAACTgCAGTTTCATCATCATCATTTAAGCAATAAACTGTTTC |
| **Ku80-E304R-F** | GATGATGATGAAACTcgAGTTTTAAAAGAGGATATTATTCAAGGGTTCCGC |
| **Ku80-E304R-R** | CCTCTTTTAAAACTcgAGTTTCATCATCATCATTTAAGCAATAAACTGTTTC |
| **mCh-Kpn2-F** | ctcgtcTCCGGAGCCACCATGGTGAGCAAGGGCGAGGAGG |
| **mCh-Mlu-R** | ctcctcACGCGTCTTGTACAGCTCGTCCATGCCGCC |
| **mCh-Xba-F** | cctctgTCTAGAgTCCAAGGGCGAAGAAGATAATATGGC |
| **mCh-Xho-R** | cctgtgCTCGAGcaTCACTTGTAAAGTTCGTCCATTCCACC |
| **mTagBFP-Acc65-F** | ctgtctGGTACCTGGAAGTGGAAGCCCAAAGAAAAAGC |
| **mTagBFP-Mlu-R** | ctgtcgACGCGTTTCAATTAAGCTTGTGCCCCAGTTTGCTAGGG |
| **PAXX-Mlu-F** | ctcctgACGCGTTCTGGTAGCGGTTCAGGACTCAGATCCATGGACCCGCTGTC |
| **PAXX-Bcu-R** | cctctcACTAGTTTAGGTCTCATCGAAGTCCACGCCACCAG |
| **PolL-Mlu-F** | ctcctcACGCGTATGGAcCCCAGGGGTATCTTGAAGGCATTTC |
| **PolL-Bcu-R** | cctctcACTAGTTCACCAGTCCCGCTCAGCAGGTTC |
| **PolL-BRCT-Bcu-R** | cctgacACTAGTtcaACTGGGGATGAAGATGCTGAATCCAGCTACATC |
| **PolL-dead-F** | GGCGACCTGTGGTGcTGTCGcCGTGCTCATCACTCACCCAGATGGCC |
| **PolL-dead-R** | GAGTGATGAGCACGgCGACAgCACCACAGGTCGCCTTTCCCCGTC |
| **PolL-R57A-F** | CATTGGACGAGCCgcGGCAGAACTCTTTGAGAAGCAGATTGTTC |
| **PolL-R57A-R** | CAAAGAGTTCTGCCgcGGCTCGTCCAATGCCAGTGCGCACAAC |
| **PolL-R57E-F** | CATTGGACGAGCCgaGGCAGAACTCTTTGAGAAGCAGATTGTTC |
| **PolL-R57E-R** | CAAAGAGTTCTGCCtcGGCTCGTCCAATGCCAGTGCGCACAAC |
| **PolL-L60A-F** | CCGGGCAGAAgcCTTTGAGAAGCAGATTGTTCAGCATGGC |
| **PolL-L60A-R** | CTGCTTCTCAAAGgcTTCTGCCCGGGCTCGTCCAATGCCAGTGC |
| **PolL-L60R-F** | CCCGGGCAGAACgCTTTGAGAAGCAGATTGTTCAGCATGGC |
| **PolL-L60R-R** | CTGCTTCTCAAAGcGTTCTGCCCGGGCTCGTCCAATGCCAGTGC |
| **PolL-F61G-F** | CGGGCAGAACTCggTGAGAAGCAGATTGTTCAGCATGGC |
| **PolL-F61G-R** | CAATCTGCTTCTCAccGAGTTCTGCCCGGGCTCGTCCAATGC |
| **PolL-R96A-F** | GAGCGAGCCCTCgcCCTTCTCAGACTACCCCAGCTGCC |
| **PolL-R96A-R** | GTCTGAGAAGGgcGAGGGCTCGCTCATAGTCCATGCC |
| **PolL-S116G-F** | GAAGTCAGCCTGGCTGgGCTTGTGCCTTCAGGAGAGGAGGCTGG |
| **PolL-S116G-R** | CCTGAAGGCACAAGCcCAGCCAGGCTGACTTCACCAGC |
| **PolM-Mlu-F** | GAATCCTGGACCCGGGtcactcACGCGTATGTTGCCAAAGAGACGCAGGGCCAGGGTC |
| **PolM-Bcu-R** | CCAGAGGTTGATTATCATATGACTAGTTCAAGCGTTACGCTGTTCAGGGGGC |
| **PolM-R43A-F** | CGTATGGGAAGAAGCgcTCGCGCTTTCCTCACCGGATTGGCCAGGTC |
| **PolM-R43A-R** | CGGTGAGGAAAGCGCGAgcGCTTCTTCCCATACGCGGCTCAACC |
| **PolM-F46A-F** | GCCGTCGCGCTgcCCTCACCGGATTGGCCAGGTCAAAGGGTTTC |
| **PolM-F46A-R** | GCCAATCCGGTGAGGgcAGCGCGACGGCTTCTTCCCATACGCGGCTC |
| **PolM-L50A-F** | CCTCACCGGAgcGGCCAGGTCAAAGGGTTTCAGAGTGCTCGAC |
| **PolM-L50A-R** | CCCTTTGACCTGGCCgcTCCGGTGAGGAAAGCGCGACGGCTTCTTCCC |
| **PolM-T106A-F** | CATCTCATGGCTCgCAGAGTCGTTGGGCGCTGGACAGCCAGTTCC |
| **PolM-T106A-R** | CGCCCAACGACTCTGcGAGCCATGAGATGTCGAGCAGAGCAGG |


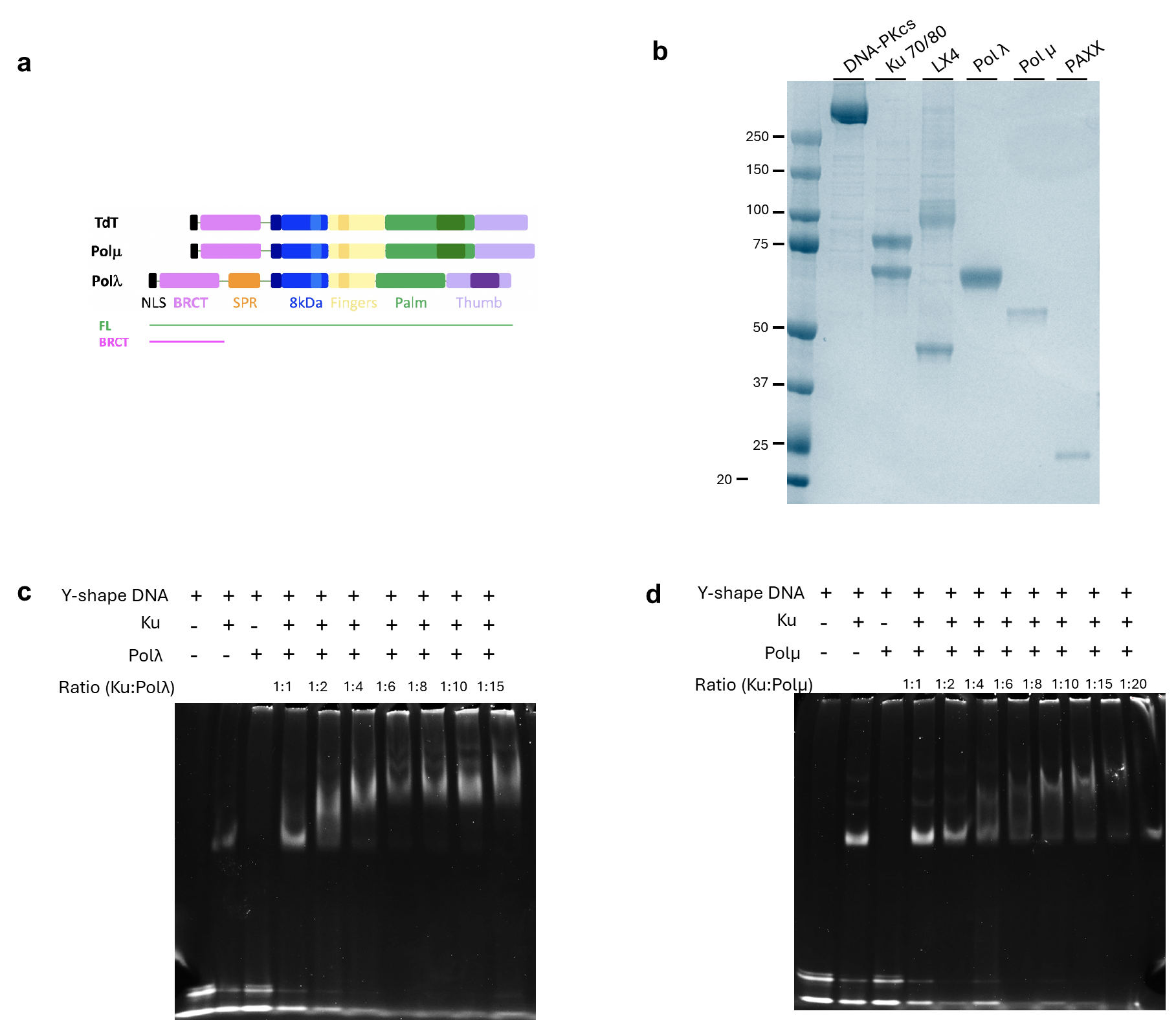


**Supplementary Figure 1. Protein purification and binding assays. a/ Domain organization of Pol X family DNA polymerases.** The Pol λ constructs used in this study correspond to the full-length protein (FL) and the amino-terminal region (residues 1-136) containing the nuclear localization sequence (NLS) and the BRCT domain (BRCT). **b)** SDS-PAGE gel analysis of DNA-PKcs, Ku70/80, LX4, Pol λ, μ and PAXX. **c)** EMSA gel analysis of Ku70/80 binding DNA and increasing concentrations of Pol λ. **d)** EMSA gel analysis of Ku70/80 binding DNA and increasing Pol μ concentrating.

**
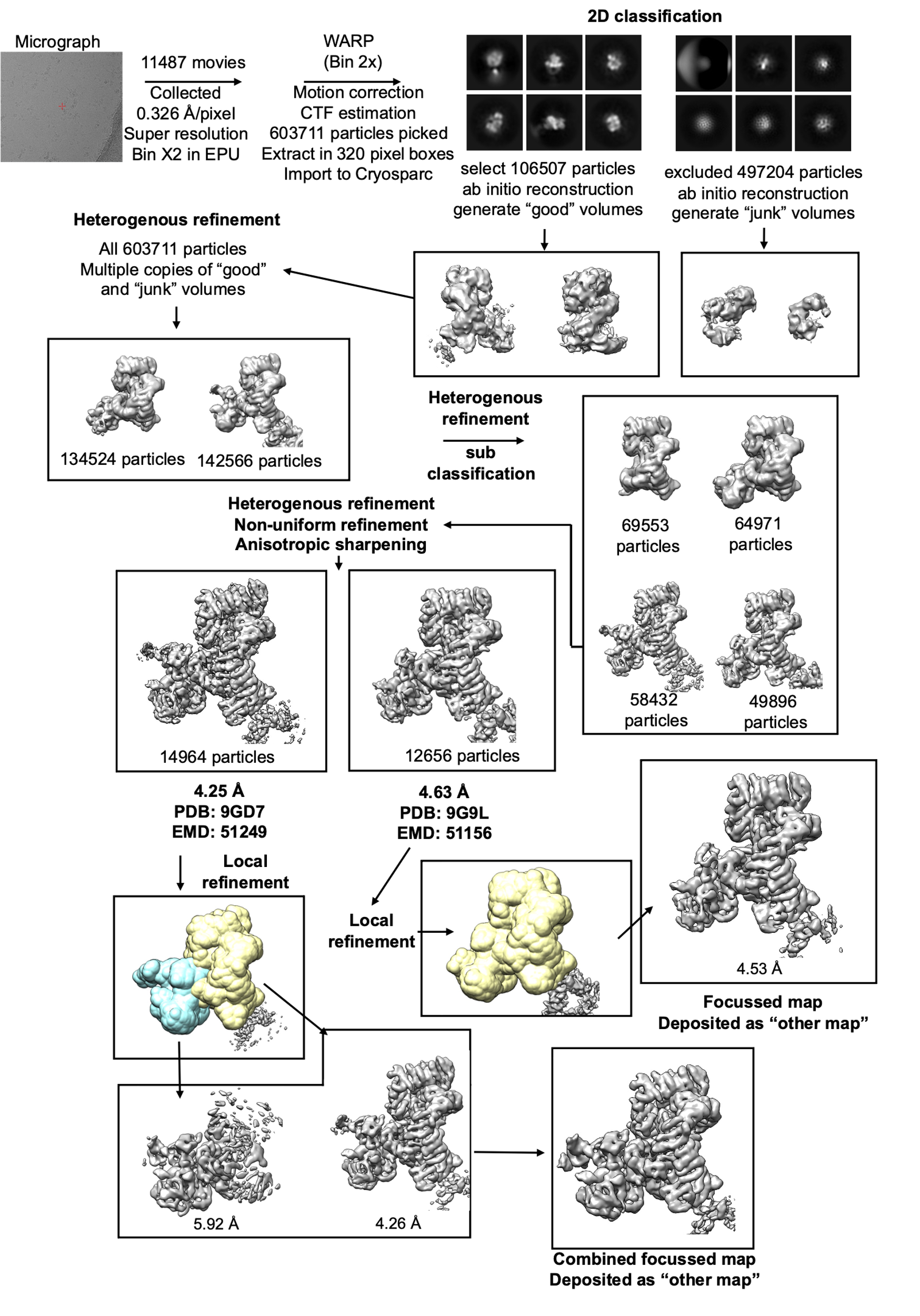
**

**Supplementary Figure 2. Single-particle cryo-EM image processing workflow for DNA-PK +PAXX + Pol λ with and without LX4.** Schematic showing particle picking using WARP and processing including 2D classification and *ab initio* reconstruction using CryoSPARC. Example micrograph and 2D class averages shown. The two main classes generated with the corresponding number of particles is shown and the two maps following non-uniform refinement with resolutions for an FSC of 0.143 are given. Additional focused and composite maps are also shown.


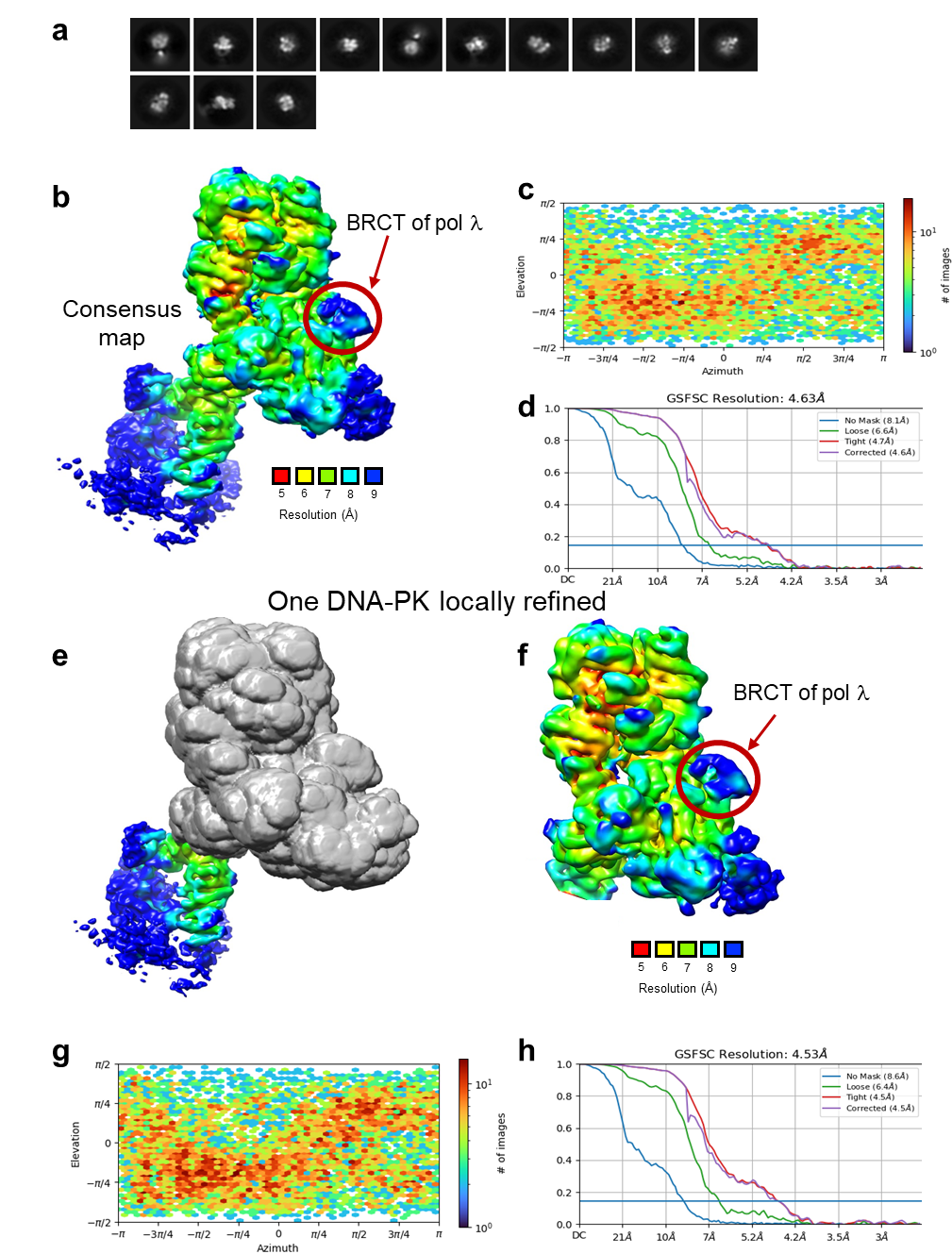


**Supplementary Figure 3.** **Cryo-EM data of DNA-PK with BRCT domain of Pol λ**. **a)** Example of 2d classes. **b)** Local resolution map of DNA-PK dimer with BRCT domain of Pol λ consensus cryo-EM map. **c)** Angular distribution calculated in cryoSPARC for particle projections shown as a heat map of the consensus map. **d)** FSC resolution curves and viewing distribution plot of the consensus map. **e)** DNA-PK dimer with BRCT domain of Pol λ consensus cryo-EM map with masking area. **f)** Local resolution map of DNA-PK dimer with BRCT domain of Pol λ locally refined map. **g)** Angular distribution calculated in cryoSPARC for particle projections shown as a heat map of the locally refined map. **h)** FSC resolution curves and viewing distribution plot of the locally refined map. The colours corresponding to each resolution are displayed on the specific key chart below the maps.


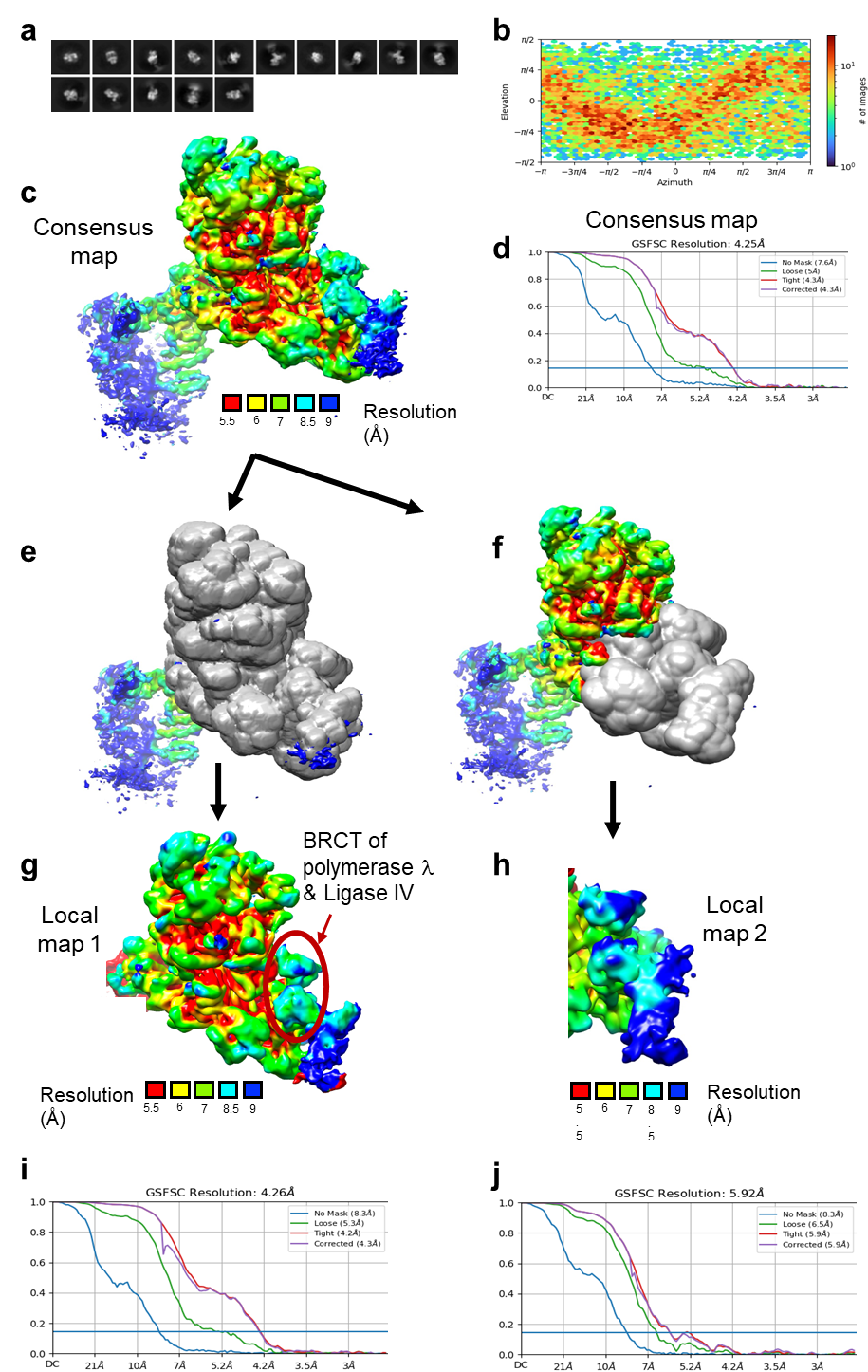


**Supplementary Figure 4. Cryo-EM data of DNA-PK with BRCT domain of Pol λ** **and LX4**. **a)** Example of 2d classes. **b)** Angular distribution calculated in cryoSPARC for particle projections shown as a heat map of the consensus map. **c)** Local resolution map of DNA-PK dimer with BRCT domain of Pol λ and Ligase IV consensus cryo-EM map. **d)** FSC resolution curves and viewing distribution plot of the consensus map. **e)** and **f)** Consensus map with masking area 1 and 2, respectively. **g)** and **h)** Local resolution of the two locally refined maps. i**)** and **j)** FSC resolution curves and viewing distribution plot of local maps 1 and 2. The colours corresponding to each resolution are displayed on the specific key chart below the maps.


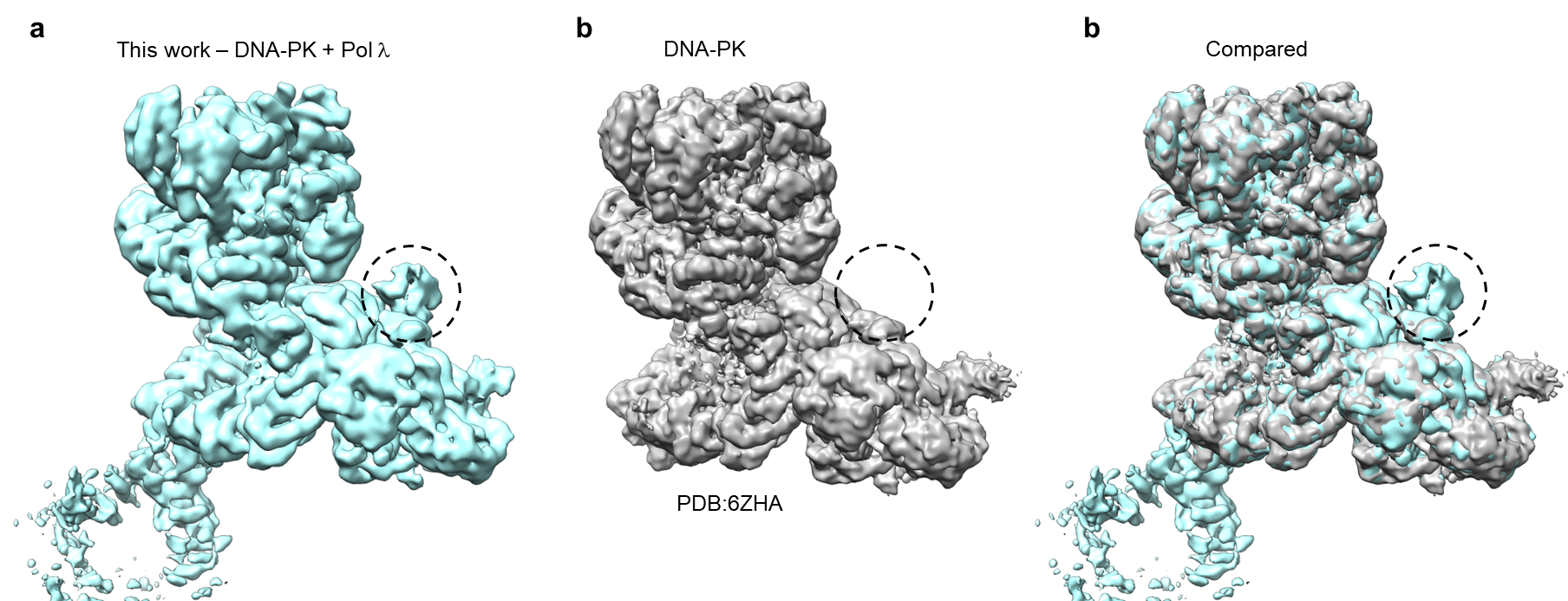


**Supplementary Figure 5. Comparison of cryo-EM maps.** **a)** DNA-PK + Pol λ (this work) in blue. **b)** DNA-PK (PDB: 6ZHA), **c)** Comparison overlay of DNA-PK + Pol λ (blue) and DNA-PK (grey).


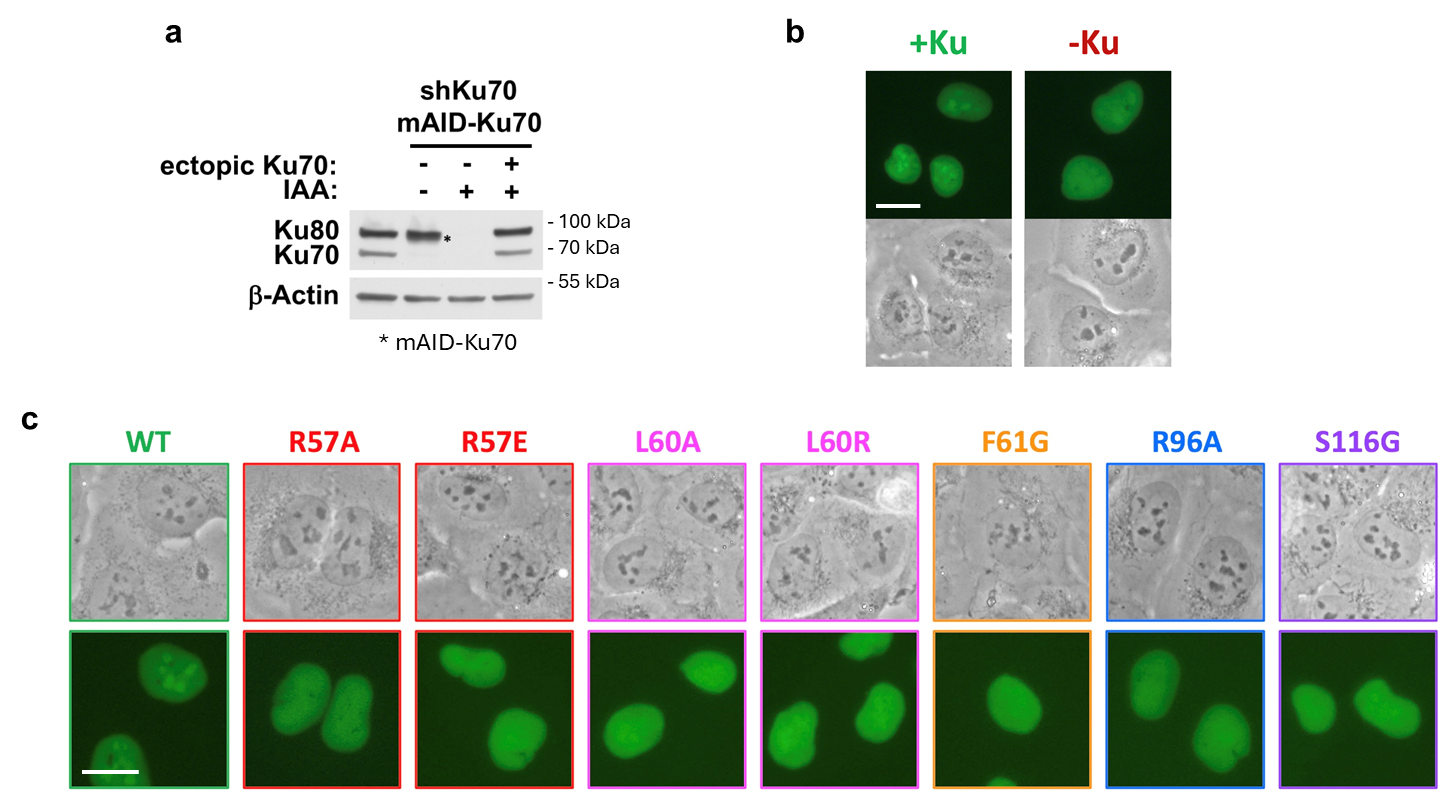


**Supplementary Figure 6. Subcellular localization of Pol**λ**full-length protein and BRCT domain. a)** Western blot on whole cell protein extracts from U2OS cells either unmodified (lane 1) or constitutively expressing an shRNA against endogenous Ku70 and rescued with expression of mAID-tagged Ku70 (lanes 2-4), treated or not with auxin (IAA) for 16 h. Asterisk indicates the position of mAID-Ku70 signal (MW: 77.4 kDa) below that of Ku80. **b)** Fluorescence micrographs of U2OS/mAID-Ku70 cells (see (a)) expressing full-length GFP-tagged Pol λ, in the presence (+Ku) or the absence (-Ku) of Ku. Scale bar represents 20 µm. **c)** Fluorescence micrographs of U2OS cells expressing WT or mutated GFP-tagged Pol λ BRCT domain. Scale bar represents 20 µm.


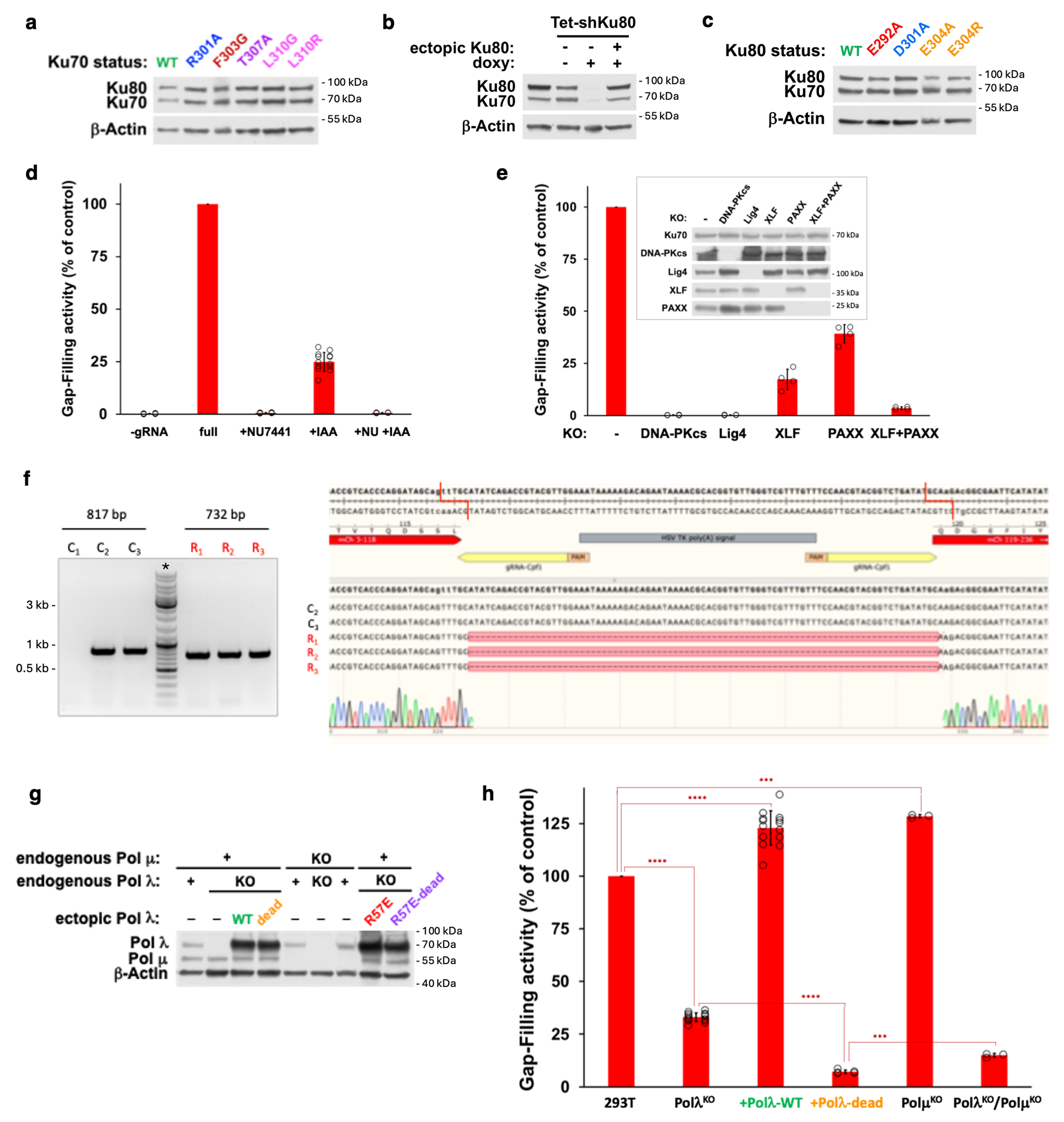


**Supplementary Figure 7. Dependency of gap-filling activity on NHEJ factors and PolX DNA polymerases.** **a)** Western blot on whole cell protein extracts from U2OS/mAID-Ku70 cells (see Suppl. Figure S6A) depleted of endogenous Ku70 and complemented with ectopic expression of either WT or mutated forms of Ku70, as indicated. **b)** Western blot on whole cell protein extracts from U2OS cells either unmodified (lane 1) or expressing a doxycycline (doxy)-induced shRNA against endogenous Ku80 (lanes 2-4) and rescued with expression of Ku80 (lane4). **c)** Western blot on whole cell protein extracts from U2OS/Tet-shKu80 cells (see (b)) depleted of endogenous Ku80 and complemented with ectopic expression of either WT or mutated forms of Ku80, as indicated. **d)** Gap-filling activity assessed in HEK-293T/mAID-Ku70 cells knocked-down for Ku70 when indicated (+IAA), in the presence or not of 3 µM DNA-PK inhibitor (+NU7441 or +NU). Results are normalized to the control condition (full) and plotted as mean values of 6 to 13 independent experiments ± SD (-gRNA: n=6; full: n=13; +NU7441: n=6; +IAA: n=13; +NU+IAA: n=6). **e)** Gap-filling activity assessed in HEK-293T cells knocked-out (KO) for different NHEJ genes, as indicated. Results are normalized to the NHEJ-proficient parental HEK-293T cell line (-) and plotted as mean values of 4 to 5 independent experiments ± SD. Inset: control western blot on whole cell protein extracts from the different KO cells. **f)** DNA junction analysis following gap-filling assay. Left: agarose gel electrophoresis showing PCR products amplified around the junction following stable genome integration of the reporter substrate and gap-filling reaction (see the Materials and methods section). Three positive red-fluorescent clones (R1, R2 and R3) and three non-fluorescent clones as a control (C1, C2 and C3) were analyzed. Expected lengths of the PCR fragments are indicated, as well as the sizes of the main DNA ladder fragments. Asterisk: GeneRuler^TM^ DNA Ladder Mix. Right: the DNA sequences of the PCR fragments are aligned with the reporter substrate sequence using the Snapgene^®^ software (Dotmatics). **g)** Western blot on whole cell protein extracts from HEK-293T cells knocked-out (KO) for POLL, POLM or both genes. When indicated, POLL KO cells were complemented with expression of ectopic WT or catalytic dead Pol λ. **h)** Gap-filling activity assessed in HEK-293T cells knocked-out (KO) for *POLL*, *POLM* or both genes. Results are normalized to the parental HEK-293T cell line and plotted as mean values of 3 to 14 independent experiments ± SD (293T: n=; 17; KO Polλ : n=14; KO Polλ + Polλ-WT: n=14; KO Polλ + Polλ-dead: n=5; KO Polμ: n=3; KO Polλ+Polμ: n=3). P-values from Student’s t-test between the indicated conditions are as follows: 293T versus KO Polλ (<0.0001 ****), 293T versus KO Polλ + Polλ-WT (<0.0001 ****), 293T versus KO Polμ (0.0002 ***), KO Polλ versus KO Polλ + Polλ-dead (<0.0001 ****), KO Polλ+Polμ versus KO Polλ + Polλ-dead (0.0001 ***). Individual values are shown as open circles. Source data are provided as a Source Data file.


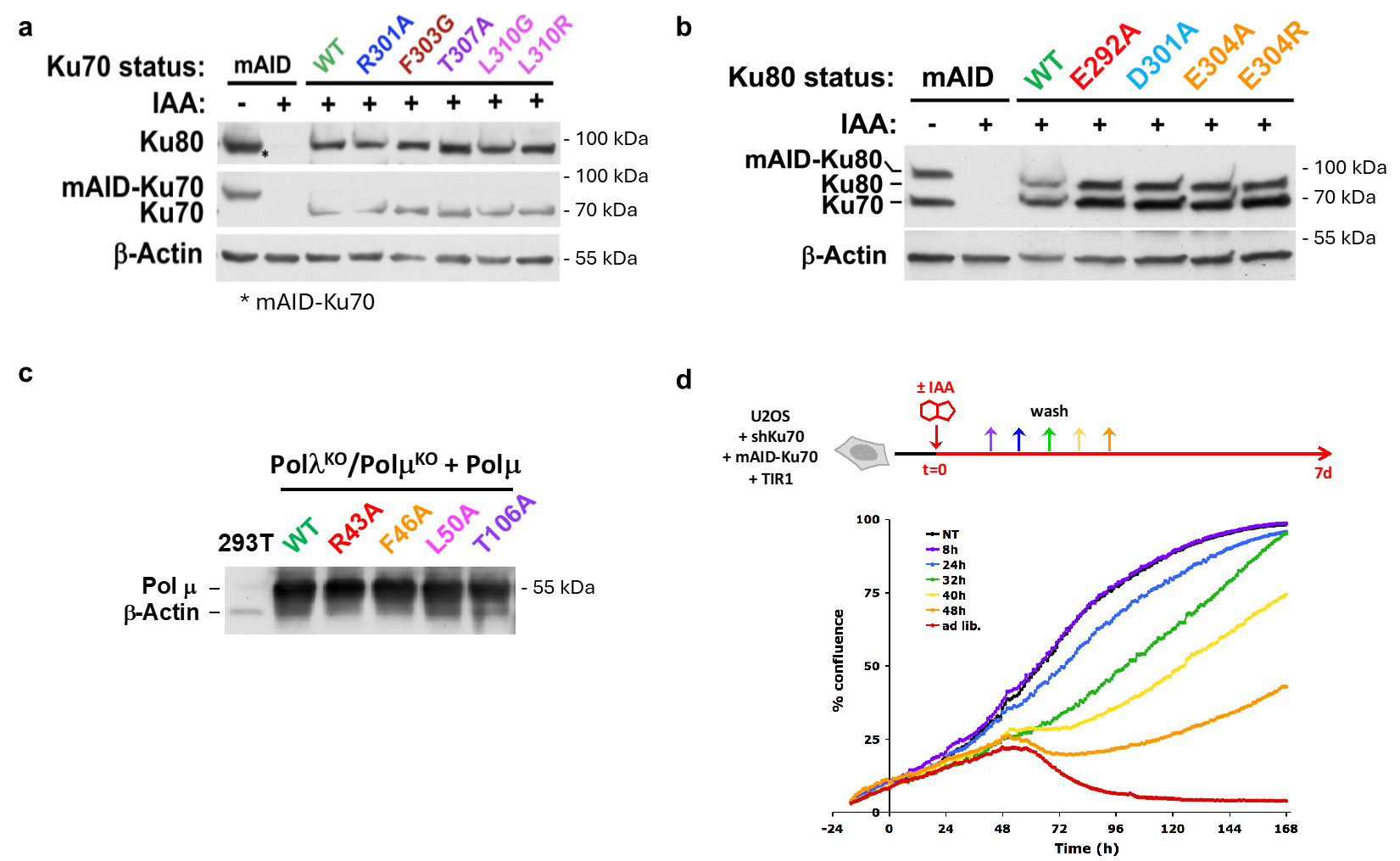


**Supplementary Figure 8. Complementation of Ku70-, Ku80- and PolX-depleted cells and time-dependent reversibility of cell lethality upon auxin-induced Ku depletion. a)** Western blot on whole cell protein extracts from HEK-293T/mAID-Ku70 cells depleted of endogenous Ku70 in the presence of auxin (+IAA) and rescued with ectopic expression of either WT or mutated forms of Ku70, as indicated. Asterisk indicates the position of mAID-Ku70 signal just below that of Ku80, which persisted after previous hybridization of the membrane with anti-Ku70 antibody. **b)** Western blot on whole cell protein extracts from HEK-293T/mAID-Ku80 cells depleted of endogenous Ku80 in the presence of auxin (+IAA) and rescued with ectopic expression of either WT or mutated forms of Ku80, as indicated. **c)** Western blot on whole cell protein extracts from parental HEK-293T cells or knocked-out (KO) for both *POLL* and *POLM* genes. When indicated, KO cells were complemented with expression of ectopic WT or mutant Polμ. **d)** HEK-293T/mAID-Ku70 cells were seeded in 12-well plates and treated with auxin (+IAA) for the indicated time. Cell proliferation was then analyzed continuously up to 7 days by assessing confluence with an IncuCyte-ZOOM (Essen Bioscience). Source data are provided as a Source Data file.


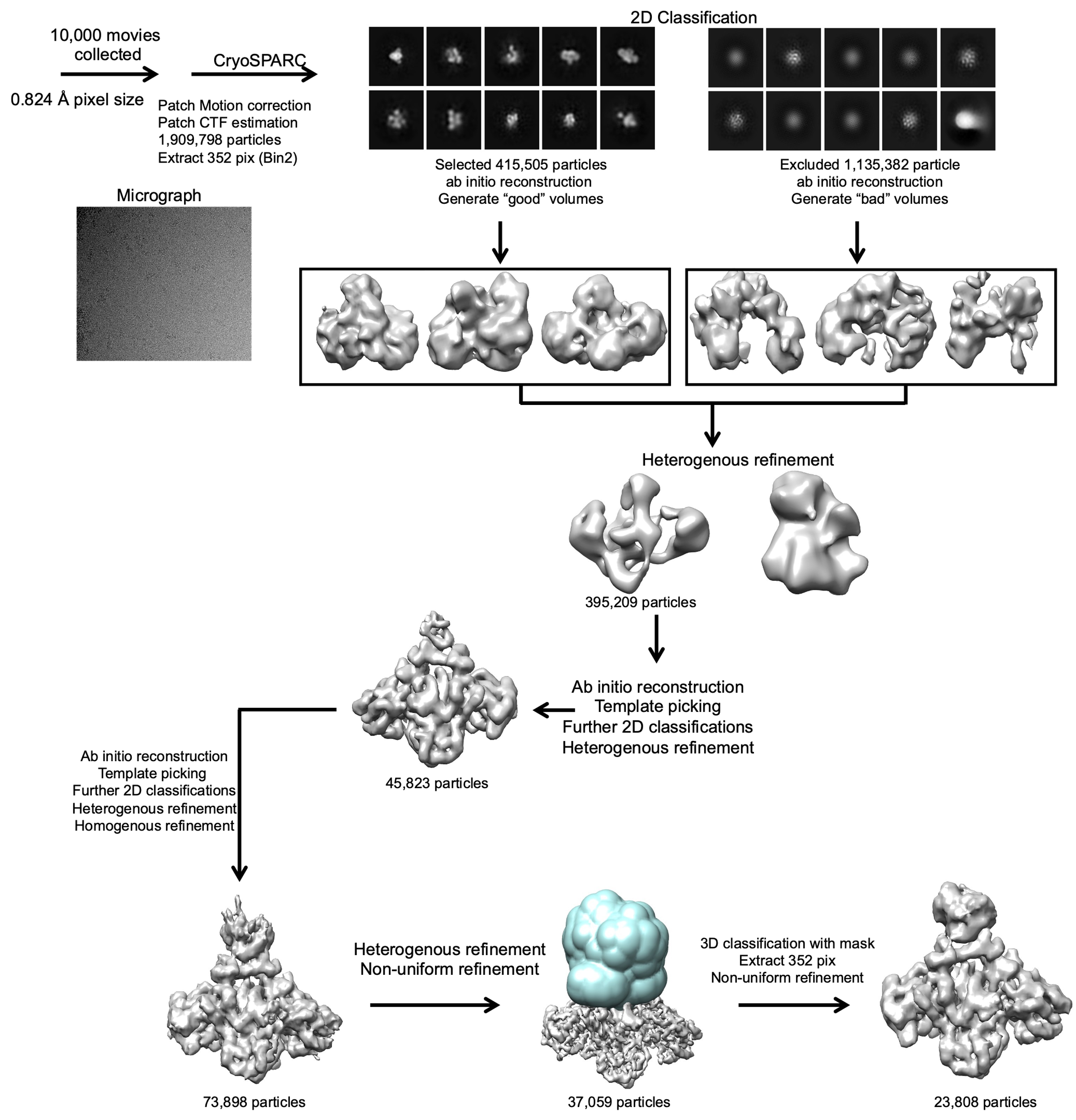


**Supplementary Figure 9: Single-particle cryo-EM image processing workflow for Ku70/80-DNA with Pol μ.** Schematic showing particle picking, 2D classification and *ab initio* reconstruction using CryoSPARC. The main class generated with the corresponding number of particles is shown and the map following non-uniform refinement with resolutions for an FSC of 0.143 are given. Mask of BRCT domain shown and 3D classification before final refinement.


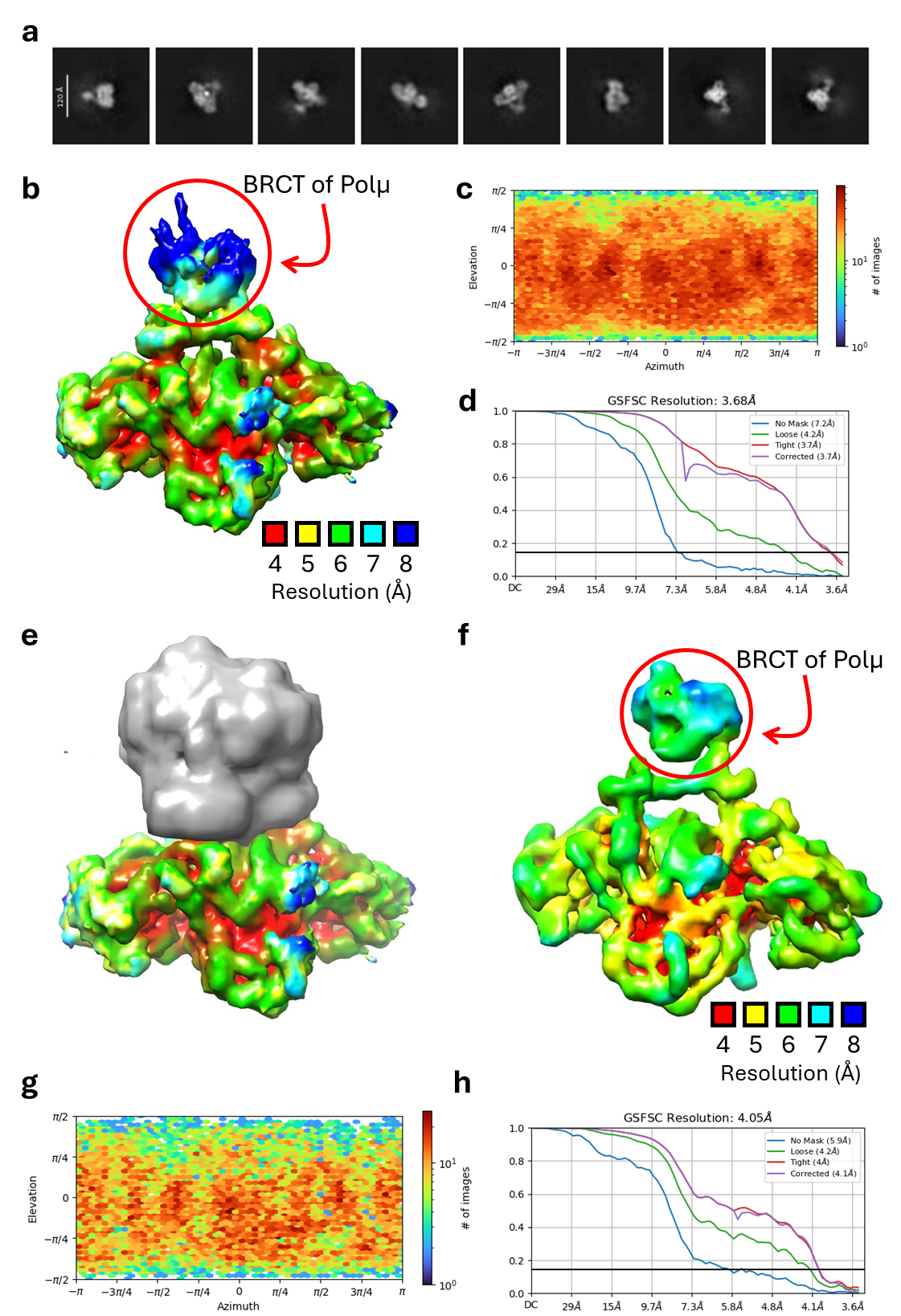


**Supplementary Figure 10. Cryo-EM data of Ku70/80-DNA with Polμ. a)** Example 2D Class averages. **b)** Local resolution map of the consensus map with resolution in key chart. **c) V**iewing distribution plot of the consensus map. **d)** FSC resolution curves. **e)** Mask for BRCT domain of Polμ. **f)** Local resolution map for the final refined map. **g) V**iewing distribution plot of the final map. **h)** Final FSC resolution curves


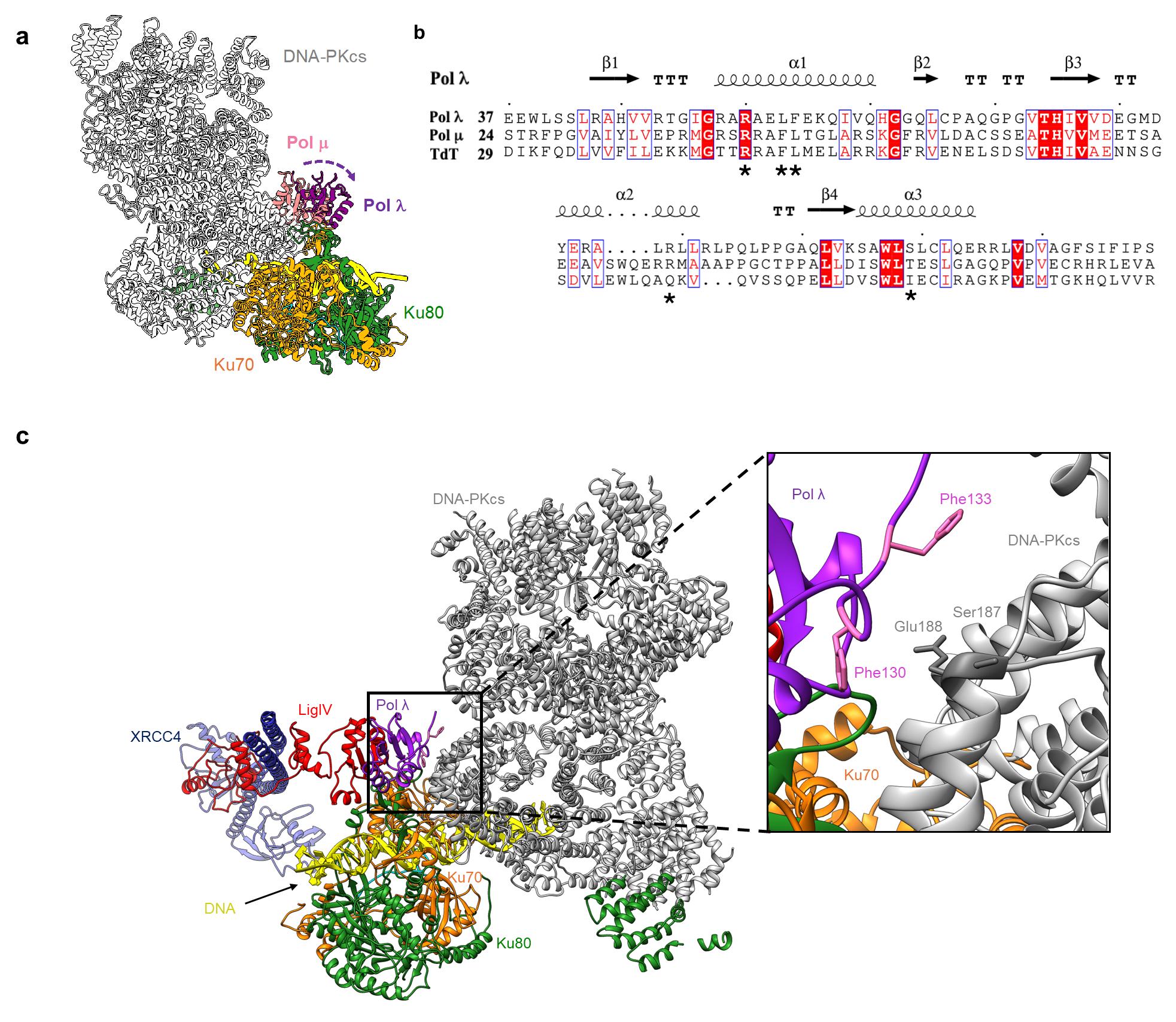


**Supplementary Figure 11.** **a)** Comparison of Pol λ within the long-range complex with Pol μ in Ku70/80, showing how Pol μ would clash with DNA-PKcs. **b)** Sequence alignment of the BRCT domain of Pol X family members generated by ESPript. Structural elements from the Pol λ cryo-EM structures are shown above the alignment. Residues mutated in this study are indicated with *. **c)** Interaction between Pol λ and DNA-PKcs. Inset, close up view of interaction between Pol λ and DNA-PKcs with residues potentially involved. DNA-PKcs is in grey, Ku70 in orange, Ku80 in green, LigIV in red, DNA yellow, XRCC4 in blue and Pol λ in purple.


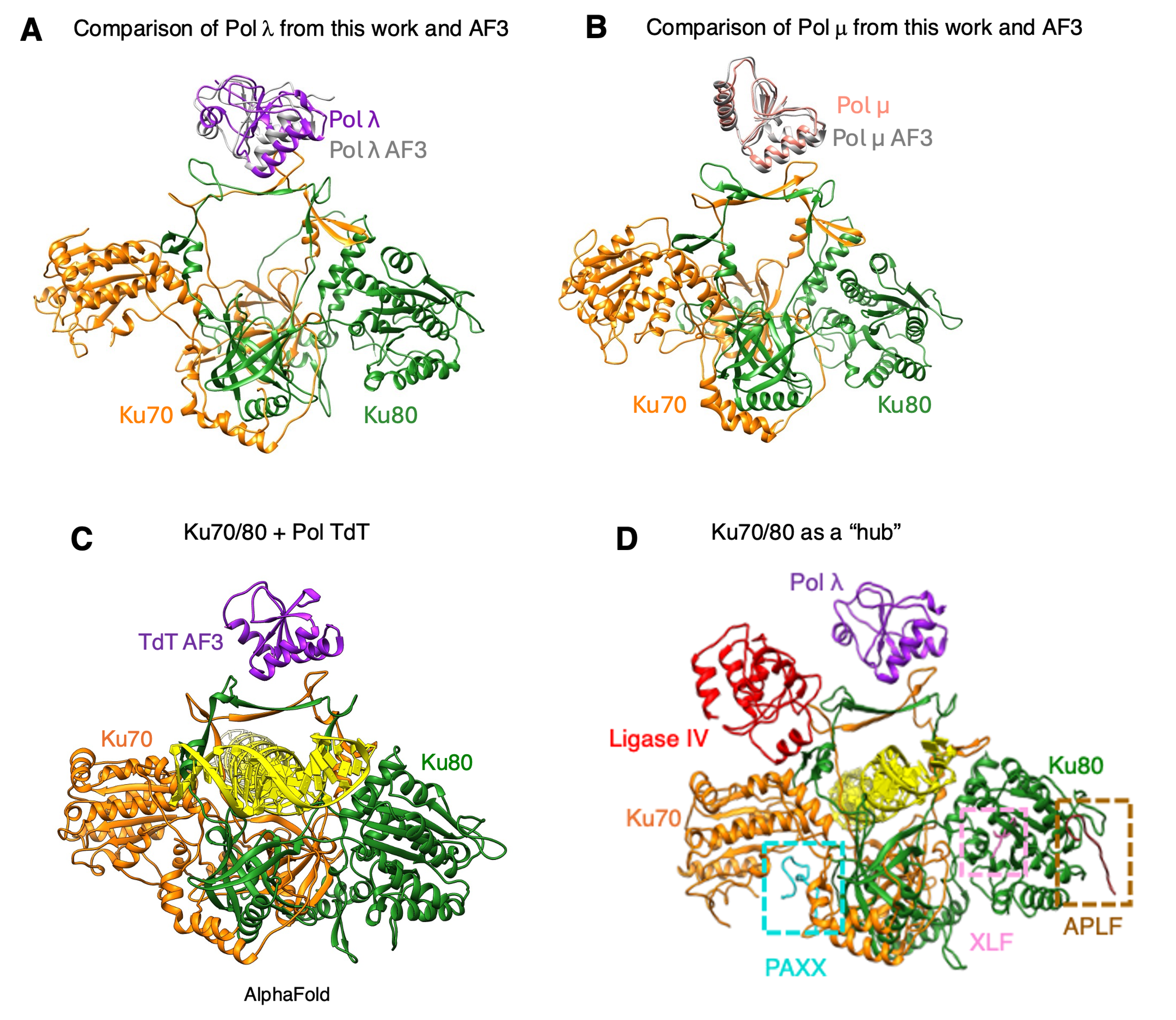


**Supplementary Figure 12.** **BRCT domains of Pol X family binding to Ku70/80. a)** Comparison for Pol λ (purple) from the long-range complex bound to Ku70/80 with Pol λ from AF3 (grey). **b)** Comparison for Pol μ (pink) from our data with Ku70/80 with AF3 Pol μ (grey). Ku70 in orange, Ku80 in green. **c)** AlphaFold 3 prediction of Pol TdT binding to Ku70/80 and DNA. Ku70 is in orange, Ku80 in green, DNA in yellow, PAXX P-KBM in cyan and Polymerase BRCTs in purple. **d)** Ku70/80 (orange/green) as a structural hub showing the binding of Pol λ (purple), Ligase IV (red), PAXX (cyan), XLF (pink) and APLF (brown).


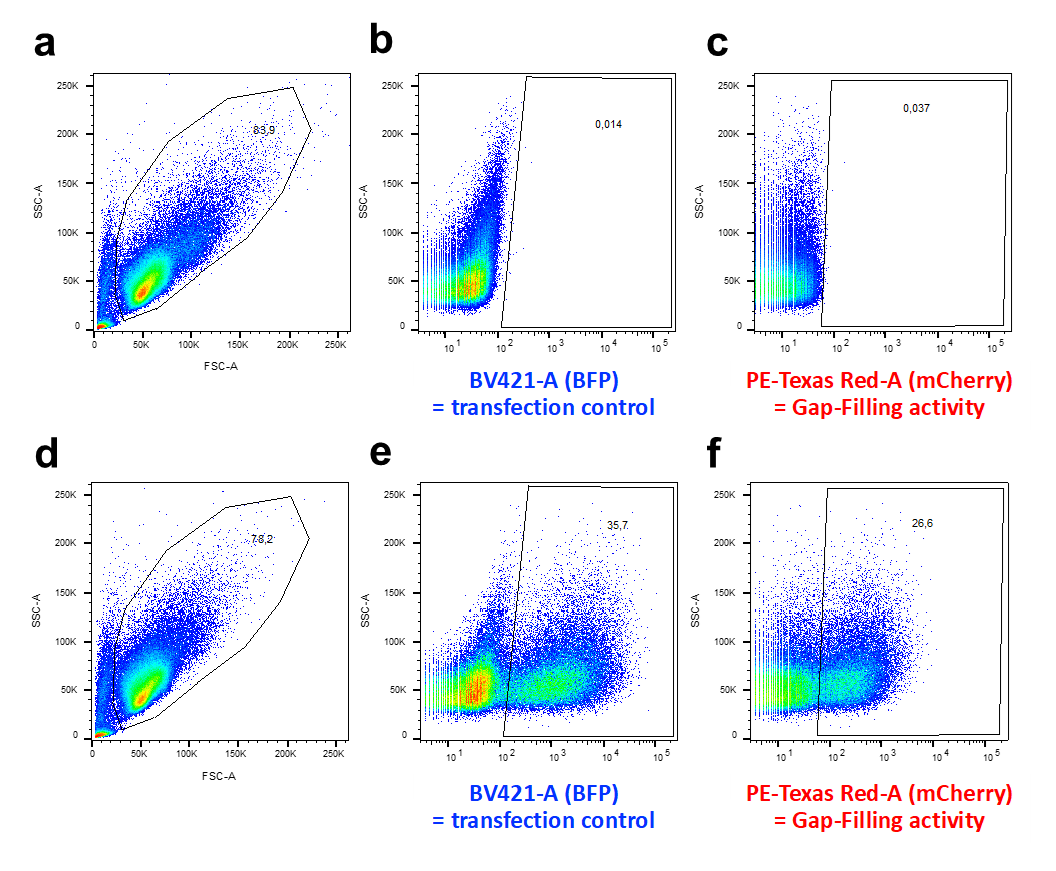


**Supplementary Figure 13. Flow cytometry gating strategy used for the Gap-Filling assay. a)** Cell debris and dead cells were excluded based on forward scatter area (FSC-A) and side scatter area (SSC-A). Gated FSC/SSC population of untransfected cells (a) was used to position boundaries between negative and positive cells for blue (BV421-A) (**b**) and red (PE-Texas Red-A) fluorescence (**c**). The same gates were applied to transfected cells (**d**, **e** and **f**) to measure blue and red fluorescence accounting for transfection efficiency and gap-filling repair activity, respectively.
