# Supplementary material for: Structural and functional insights into the interaction between Ku70/80 and Pol X family polymerases in NHEJ
Source: Nat Commun. 2025 May 6;16:4208. doi: 10.1038/s41467-025-59133-2 (PMC12056208; doi:10.1038/s41467-025-59133-2)
Supplement: Supplementary file 2 — Reporting Summary [file 41467_2025_59133_MOESM2_ESM.pdf]

nature portfolio

Corresponding author(s): Dr Amanda K Chaplin & Dr Patrick Calsou

Last updated by author(s): Feb 19, 2025

Reporting Summary

Nature Portfolio wishes to improve the reproducibility of the work that we publish. This form provides structure for consistency and transparency in reporting. For further information on Nature Portfolio policies, see our Editorial Policies and the Editorial Policy Checklist.

Statistics

For all statistical analyses, confirm that the following items are present in the figure legend, table legend, main text, or Methods section.

n/a	Confirmed	
☐	☒	The exact sample size (*n*) for each experimental group/condition, given as a discrete number and unit of measurement
☐	☒	A statement on whether measurements were taken from distinct samples or whether the same sample was measured repeatedly
☐	☒	The statistical test(s) used AND whether they are one- or two-sided<br>*Only common tests should be described solely by name; describe more complex techniques in the Methods section.*
☒	☐	A description of all covariates tested
☒	☐	A description of any assumptions or corrections, such as tests of normality and adjustment for multiple comparisons
☐	☒	A full description of the statistical parameters including central tendency (e.g. means) or other basic estimates (e.g. regression coefficient) AND variation (e.g. standard deviation) or associated estimates of uncertainty (e.g. confidence intervals)
☐	☒	For null hypothesis testing, the test statistic (e.g. *F*, *t*, *r*) with confidence intervals, effect sizes, degrees of freedom and *P* value noted<br>*Give P values as exact values whenever suitable.*
☒	☐	For Bayesian analysis, information on the choice of priors and Markov chain Monte Carlo settings
☒	☐	For hierarchical and complex designs, identification of the appropriate level for tests and full reporting of outcomes
☒	☐	Estimates of effect sizes (e.g. Cohen's *d*, Pearson's *r*), indicating how they were calculated

Our web collection on statistics for biologists contains articles on many of the points above.

Software and code

Policy information about availability of computer code

Data collection	Data from multiphoton laser micro-irradiation were collected on an LSM710 microscope (Zeiss) using the ZEN interface<br>Repair activity data from flow cytometry analysis were collected on a Fortessa X-20 cell analyzer (BD Biosciences)
Data analysis	Data were analyzed with the following softwares: Microsoft Excel 16.16, FlowJo v10, GraphPad calculators for statistical analysis (https://www.graphpad.com/quickcalcs/contMenu/), Snapgene v4.0.8 (DNA sequences alignments)

For manuscripts utilizing custom algorithms or software that are central to the research but not yet described in published literature, software must be made available to editors and reviewers. We strongly encourage code deposition in a community repository (e.g. GitHub). See the Nature Portfolio guidelines for submitting code & software for further information.

Data

Policy information about availability of data

All manuscripts must include a data availability statement. This statement should provide the following information, where applicable:

- Accession codes, unique identifiers, or web links for publicly available datasets
- A description of any restrictions on data availability
- For clinical datasets or third party data, please ensure that the statement adheres to our policy

Structural data is available at the PDB and EMDB.

Research involving human participants, their data, or biological material

Policy information about studies with human participants or human data. See also policy information about sex, gender (identity/presentation), and sexual orientation and race, ethnicity and racism.

Reporting on sex and gender	n/a
Reporting on race, ethnicity, or other socially relevant groupings	n/a
Population characteristics	n/a
Recruitment	n/a
Ethics oversight	n/a

Note that full information on the approval of the study protocol must also be provided in the manuscript.

Field-specific reporting

Please select the one below that is the best fit for your research. If you are not sure, read the appropriate sections before making your selection.

☒ Life sciences ☐ Behavioural & social sciences ☐ Ecological, evolutionary & environmental sciences

For a reference copy of the document with all sections, see nature.com/documents/nr-reporting-summary-flat.pdf

Life sciences study design

All studies must disclose on these points even when the disclosure is negative.

Sample size	No sample size calculation was performed a priori
Data exclusions	No data were excluded from the analyses
Replication	Replication was performed and reproducibility of changes was assessed using indicated statistical methods
Randomization	Randomization was not relevant in our studies
Blinding	Blinding was not relevant in our studies

Reporting for specific materials, systems and methods

We require information from authors about some types of materials, experimental systems and methods used in many studies. Here, indicate whether each material, system or method listed is relevant to your study. If you are not sure if a list item applies to your research, read the appropriate section before selecting a response.

Materials & experimental systems

n/a	Involved in the study	
☐	☒	Antibodies
☐	☒	Eukaryotic cell lines
☒	☐	Palaeontology and archaeology
☒	☐	Animals and other organisms
☒	☐	Clinical data
☒	☐	Dual use research of concern
☒	☐	Plants

Methods

n/a	Involved in the study	
☒	☐	ChIP-seq
☐	☒	Flow cytometry
☒	☐	MRI-based neuroimaging

Antibodies

Antibodies used	Primary antibodies used:<br>- mouse monoclonal anti-DNA-PKcs antibody: clone 18.2 from Thermo Fisher Scientific, MA5-13238, lot: Y14047369, (https://www.thermofisher.com/antibody/product/DNA-PK-Antibody-clone-18-2-Monoclonal/MA5-13238)<br>- mouse monoclonal anti-Ku80 antibody: clone 111 from Thermo Fisher Scientific, MA5-12933, lot: WB31922872, (https://

www.thermofisher.com/antibody/product/Ku80-Antibody-clone-111-Monoclonal/MA5-12933)
- mouse monoclonal anti-Ku70 antibody: clone N3H10 from Thermo Fisher Scientific, MA5-13110, lot: WE27669780, (https://www.thermofisher.com/antibody/product/Ku70-Antibody-clone-N3H10-Monoclonal/MA5-13110)
- mouse monoclonal anti-Pol lambda antibody: clone E11 from Santa Cruz, sc-373844, lot: B0818 (https://www.scbt.com/p/dna-pol-lambda-antibody-e-11?srsltid=AfmBOorLROoOrN2ILG716jJ0kFExf5A_8C7OuNNrJegJkaKtOGBRW0k3)
- mouse monoclonal anti-beta-Actin antibody: clone C4 from Santa Cruz, sc-47778, lot: A2023 (https://www.scbt.com/p/beta-actin-antibody-c4?srsltid=AfmBOortGbTbeAqFjJshQAiWoMBFUbBGUfu53xzlino42OjXIlZOxYGj)
- rabbit monoclonal anti-LigIV antibody: ref A11432 from Abclonal, lot: 4000000595 (https://static.abclonal.com/datasheet/A11432.pdf?v=1686746263)
- rabbit monoclonal anti-Pol mu antibody, ref EPR10470(B) from Abcam, lot: GR117969-3 (https://www.abcam.com/en-us/products/primary-antibodies/dna-polymerase-mu-antibody-epr10470b-ab157465)
- rabbit polyclonal anti-PAXX antibodies: ref NBP1-94172 from Novus, lot: C118006 (https://www.novusbio.com/products/paxx-antibody_nbp1-94172)
- rabbit polyclonal anti-XLF antibodies: ref A19957 from Abclonal, lot: 00040701118 (https://abclonal.com/catalog-antibodies/KOValidatedNHEJ1RabbitpAb/A19957?srsltid=AfmBOoqwI1Mzixcs_kT1I5Vhvo-fuPHYcKavxFVysFl6uriMvdhMp9TF)

Validation: Validations are available from the manufacturers (please refer to the provided link)

Eukaryotic cell lines

Policy information about cell lines and Sex and Gender in Research

Cell line source(s)	U2OS cells (human osteosarcoma cell line) were obtained from the ECACC (Salisbury, UK)<br>HEK-293T cells (human embryonic cells, CRL-3216) were obtained from the ATCC (Manassas, VA, USA)
Authentication	The cell lines have not been formally authenticated, except on the basis of morphological criteria and growth rates provided by the supplier
Mycoplasma contamination	Cells were tested negative for mycoplasma and regularly treated with Plasmocure
Commonly misidentified lines (See ICLAC register)	n/a

Plants

Seed stocks	n/a
Novel plant genotypes	n/a
Authentication	n/a

Flow Cytometry

Plots

Confirm that:

- ☐ The axis labels state the marker and fluorochrome used (e.g. CD4-FITC).
- ☐ The axis scales are clearly visible. Include numbers along axes only for bottom left plot of group (a 'group' is an analysis of identical markers).
- ☐ All plots are contour plots with outliers or pseudocolor plots.
- ☐ A numerical value for number of cells or percentage (with statistics) is provided.

Methodology

Sample preparation	HEK-293T cells from 6-well plate cultures were harvested following trypsinization, washed with PBS and further dissociated on 70 µm filters
Instrument	Data were collected on a Fortessa X-20 cell analyzer (BD Biosciences)
Software	Data were analyzed with the FlowJo v10 software
Cell population abundance	Cell population was not limiting and 100.000 cells were commonly analysed for each condition

Gating strategy

Cell debris and dead cells were excluded based on forward scatter area (FSC-A) and side scatter area (SSC-A). Gated FSC/SSC population of untransfected cells was used to position boundaries between negative and positive cells for blue (BV421-A) and red (PE-Texas Red-A) fluorescence. The same gates were applied to transfected cells to measure blue (BFP) and red (mCherry) fluorescence accounting for transfection efficiency and gap-filling repair activity, respectively. A gating example is provided in Supplementary Figure 13.

☒ Tick this box to confirm that a figure exemplifying the gating strategy is provided in the Supplementary Information.
